# Understanding the characteristics of the microenvironments in urban street canyons through analysis of pollution measured using a novel pervasive sensor array

**DOI:** 10.1007/s10661-014-3939-7

**Published:** 2014-08-10

**Authors:** Fabio Galatioto, Margaret Carol Bell, Graeme Hill

**Affiliations:** School of Civil Engineering and Geosciences, Newcastle University, Devonshire Building–Kensington Terrace, Newcastle upon Tyne, NE1 7RU UK

**Keywords:** Air quality, Pervasive sensors, Oxides of nitrogen, Cluster analysis, Street canyon, Urban area, Traffic flow regimes

## Abstract

This paper presents results of comprehensive analyses of data from the first 122 commercially available wireless environmental pervasive sensors (*motes*), developed by Newcastle University and deployed in England. Measurements of pollution, meteorology and traffic are used to investigate the complexity of the physical and chemical processes governing the levels of traffic-related pollution in urban areas. Following a brief introduction on health impacts associated with air quality, description of the mote technology is given. Cluster analysis statistics to investigate the relationship between different pollutant types and traffic data demonstrated that traffic flow regimes alone cannot be used to estimate diurnal kerbside pollutant concentrations. Also, the absolute levels, whilst dependent on meteorological conditions and static parameters are only partially governed by the pollutant dispersion. The research clearly illustrates the benefits and added value of pervasive concentration measurement in urban micro environments with potential to effectively evaluate human exposure to transport-related emissions.

## Introduction

Air pollution is a major environmental risk to health (WHO 2000). By reducing air pollution levels, we can help reduce the global burden of disease from respiratory infections, heart disease and lung cancer. Research to date has revealed evidence that higher levels of air pollution in a city have increased incident of respiratory (both long- and short-term) and cardiovascular health of the population (WHO 2000). In addition, urban outdoor air pollution is estimated to cause 1.3 million deaths worldwide per year (WHO 2010); however, the effects of air pollution have not been fully quantified. Earlier, short-term exposure studies have reported positive associations between air pollution and all-cause mortality (Schwartz and Dockery 1992; Touloumi et al. 1994; Scoggins et al. 2004), cardiovascular mortality (Morgan et al. 1998) and respiratory mortality (Lebowitz 1996; Hales et al. 2000). Epidemiological studies have shown that symptoms of bronchitis in asthmatic children increase in association with long-term exposure to nitrogen dioxide, NO_2_ (Rodrigueza et al. 2007), and more recent research suggests effects on the heart (Ackermann-Liebrich 2011). Reduced lung function growth is linked to NO_2_ at concentrations currently measured (or observed) in cities of Europe (Gotschi et al. 2008), USA (Gauderman et al. 2004) and more recently in Eastern countries (Hea et al. 2010). A quantitative research synthesis of peer-reviewed publications over last 30 years, conducted by Meng et al. (2012) on personal exposures of ambient pollutant concentrations suggests that ambient NO_2_ is a good surrogate for personal exposure and depends on various factors, including season, age of the study population, pre-existing disease and possibly indoor and local sources and sampling aspects. Exposure to air pollutants is largely beyond the control of individuals and requires action by public authorities at the national, regional and even international levels.

Most local government policy measures covering outdoor air quality aim to ensure compliance with national air quality objectives and standards for pollutants’ emissions at the source. Overall, in European background locations, the average levels of urban particulate matter, PM, did not change substantially in the period 1997–2007 (WHO 2010). Concentrations of another common urban air pollutant, NO_2_, fell more consistently, but the reduction was small. However, since 2007, evidence that NO_2_ levels are increasing, due to primary NO_2_ emissions from Euro 4 diesel vehicles and increase in number of diesel vehicles on UK roads, are emerging (DEFRA 2009; Bell et al. 2013). This contrasts with the pronounced downward trend evident for sulphur dioxide, indicating that policies aimed at the reduction of sulphur emissions, mainly burning of coal and sulphur fuels, have been more effective than those addressing particulate matter (PM) or nitrogen oxide emissions from the traffic fleet. The 2008 ambient air quality directive (2008/50/EC) replaced nearly all the previous EU air quality legislation and set legally binding limits for concentrations in outdoor air of major pollutants that impact public health such as PM_10_, PM_2.5_ and NO_2_. As well as having direct effects, NO_2_ can combine with the oxygen in the atmosphere to form secondary pollutants including ozone, O_3_, which is a harmful air pollutant (and potent greenhouse gas) which can be transported great distances by weather systems.

In 2008, 45 % of the EU’s population still lived in zones exceeding PM_10_ limit values, 49 % in zones exceeding the annual NO_2_ limit value and 46 % in zones exceeding the O_3_ health target value (EEA 2010). Given this evidence, it is clear that air quality objectives are still not fully met, and the WHO-estimated 1.3 million deaths worldwide per year are associated with urban outdoor air pollution, there is clearly much work and research still to be done. Air pollution is one of the main environmental concerns facing EU citizens. The European Commission launched, in 2011, a public consultation on its current policy in this area to identify strengths and weaknesses of the existing legislative framework to set new long-term objectives beyond 2020 and progress on its review to be reported by 2013. The 2008 Directive was made law in England through the Air Quality Standards Regulations 2010, which also incorporates the 4th Air Quality daughter directive (2004/107/EC) that sets targets for levels of certain toxic heavy metals and polycyclic aromatic hydrocarbons. In the UK, responsibility for meeting air quality limit values is devolved to the local administrations. The UK government and the devolved administrations are required under the Environment Act 1995 to produce a national air quality strategy. This was last reviewed and published in 2007.

The strategy sets out the UK’s air quality objectives and recognises that action at national, regional and local level may be needed, depending on the scale and nature of the air quality problem (DEFRA 2010). In particular, local authorities in the UK have to review air quality in their area and designate Air Quality Management Areas (AQMAs^1^) if improvements are necessary. Where an air quality management area is designated, local authorities are also required to work towards the strategy’s objectives prescribed in regulations for that purpose. An air quality action plan describing the pollution reduction measures must then be put in place. These plans contribute to the achievement of air quality limit values at a local level. In March 2010, the Department for Environment, Food and Rural Affairs (DEFRA) and the devolved administrations published the document “Air Pollution: Action in a Changing Climate”, which highlights the additional health benefits that can be achieved through closer integration of air quality and climate change policies in the future.

### Link between pollution and traffic

Road transport and industry are the main sources of pollution. Emissions from road transport, for example, are thought to account for 75 % of total UK emissions of carbon monoxide and 47 % of total emissions of oxides of nitrogen. In urban centres, the contribution from road transport is generally even higher (DEFRA 2010), because many factors such as congestion, fleet characteristics, prevailing meteorological conditions, localised transient wind fields, residential and industrial activities, etc. are contributing to air quality levels.

In the UK, for example, NO_2_ is one of the most important and monitored pollutant in urban areas, and in 2009, it was responsible for 93 % of Air Quality Management Areas, AQMAs (DEFRA 2010). However, due to the high cost of precision monitoring, it is possible only to measure pollution at a fraction of polluted areas in a city and assessing the impact of specific policies on air quality has been difficult and a complex science. Sophisticated modelling tools exist to forecast emissions from different sources; however, a rich dataset of inputs is required to ensure the accuracy of the model is not compromised, and without measurements, models cannot be calibrated and often fail to properly represent the dynamics of pollution generation. Standard monitoring systems usually involve averaging over an hour, as performed in previous studies (Maruo et al. 2003; Brown et al. 2009), a resolution that is far too long to investigate causes of pollution, the chemical and physical processes for which occur on timescales of minutes even seconds. Moreover, at a local scale, i.e. where pedestrians walk and citizens live, the variation in concentrations can be considerable and directly related to local vehicle characteristics traffic volumes, congestion levels, the shape of the built environment and orientation of the roads. For this reason, it is important to demonstrate the potential value of low-cost and easy-to-deploy pervasive sensor systems (*motes*) to monitor pollutants at high resolution synchronised with other data sources including traffic and meteorological conditions. The richer understanding of the pollutant sources which are responsible for exposure of the public in microenvironments will be made possible by the mote technology allowing the design of more effective mitigation strategies in the future.

This paper presents the results of a comprehensive analysis of simultaneous monitoring at 122 locations of traffic flow regimes (quiet, smooth flow, unstable and congested), urban concentration of nitrogen dioxide and meteorological conditions over the period October–November 2011 in an attempt to unravel the complexity in the physical and chemical processes that govern urban pollutant concentrations responsible for pollution hot-spots. The health effects of NO_2_ described above justified the importance of this research. The following section presents a description of the mote, a novel wireless monitoring system, developed at Newcastle University (Neasham et al. 2007), which has enabled pervasive monitoring of pollution at 1-min resolution over a sufficiently long period to ensure statistical significance of the results. The traffic, meteorological conditions and other data will be described before details of the case study area are presented. The steps taken in preparing the data and carrying out the cluster analysis of the synchronised datasets will be articulated before the “Results and discussions” are presented and followed by “Conclusions and future directions”.

## Materials and methods

### Pervasive sensor array

**Fig. 1:**
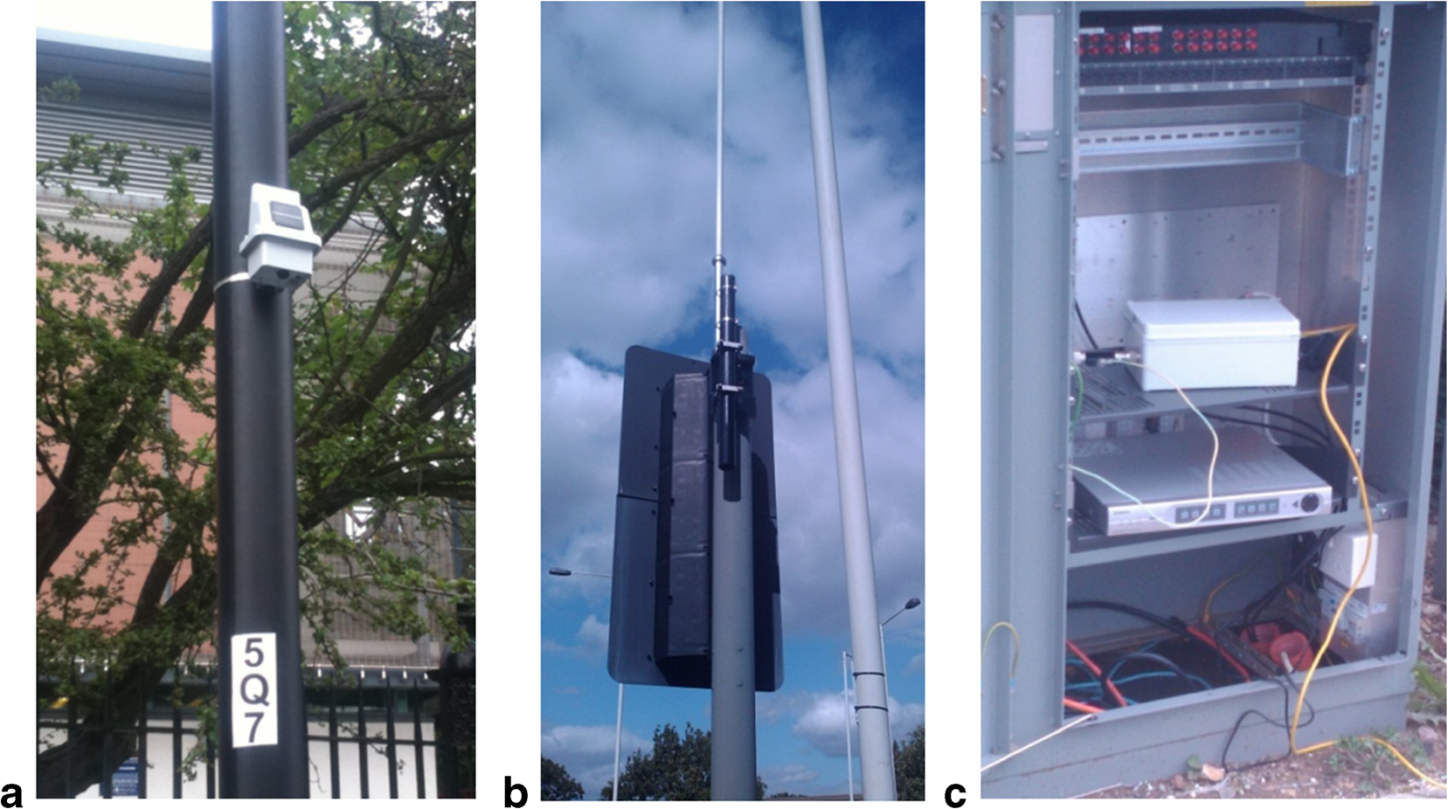
Mote mounted on a lamppost (**a**), Wi-Fi antenna to enable long-range communication (**b**) and gateway installed in a traffic light box furniture (**c**)

Although numerous commercial environmental sensor products are already on the market, (http://www.xbow.com/Products/wproductsoverview.aspx), none have the necessary sensor payload or flexibility in power management and communications required for roadside pollutant measurements. Hence, a custom-designed low-cost wireless pervasive sensor, namely a mote, was developed at Newcastle University to meet these requirements. The research was funded jointly by the Engineering and Physical Sciences Research Council and the Department for Transport, UK, in the Mobile Environmental Sensing System Across Grid Environments (MESSAGE) project (http://bioinf.ncl.ac.uk/ message/). The key drivers in the design were cost, battery life and physical size with a prime objective to establish the value and importance of data synchronisation with legacy ITS systems measurements. This new generation of low-cost wireless pervasive sensors system or mote is easily deployed on street furniture (Fig. 1a). The mote is capable of measuring multiple parameters and relaying data using a wireless communications system (Fig. 1b) via a gateway. Individual electrochemical sensors, with three electrodes (working, counter and reference electrode) within the mote, measure nitric oxide, NO, and NO_2_, both within a range of 0–500 ppb, and carbon monoxide, CO (0–10 ppm) (as an option, the CO sensor can be substituted with an O_3_ sensor, range 0–200 ppb). A low-cost microphone is used to measure noise level in decibel(A), with an adjustable 45-dB(A) range (such as 45–90 dB(A)), and ultra-low cost digital sensors for temperature (0.1 °C resolution) and relative humidity (10–100 %). The mote has a low-power microprocessor (PIC18F4620) and ZigBee wireless mesh networking which implement the IEEE 802.15.4 communication standard protocol (IEEE 2006). This allows data to be transmitted via power-efficient networking to a neighbouring mote some 80–100 m away allowing up to five hops to a gateway (Fig. 1c). The latter requires main power and uses GPRS, or an existing LAN/Internet network, to transmit the data to a central server where it is stored in the Newcastle University Integrated Database and Assessment Platform (NUIDAP, Bell et al. 2011b), which is a bespoke system constructed using open source software (Postgres).

The core component to the design is an 8-bit, low-power microprocessor with a large number of digital and analogue ports. The architecture was designed so that the processor core and some of the sensor/communications circuits can be powered down when not in use. This enables the power consumption of the motes to be precisely controlled, depending on the desired sampling rate, integration time and transmission schedule for the sensor data, to achieve a battery life of up to 1 year from the cell. By using a mobile ad-hoc network (MANET) protocol, such as AODV (Royer and Toh 1999), optimum routing is maintained as nodes move in/out of range or if nodes fail. Using this protocol, with up to 100 motes, an area of approximately 1 km^2^ is covered. An intelligent algorithm is used to reroute data via different sensors to compensate for obstructions in connectivity (caused by, for example, the passage of a heavy goods vehicle) expected in an urban site.

These sensors have the capacity to assess microenvironments not only in heavily trafficked areas but also in previously unmonitored road networks in urban and potentially inter-urban areas, such as motorways and rural roads, resulting in more realistic assessments of the causes of pollution and measurement of impacts of air quality and noise and, through the use of dose relationships, the estimation of health impact.

### Traffic monitoring

**Fig. 2:**
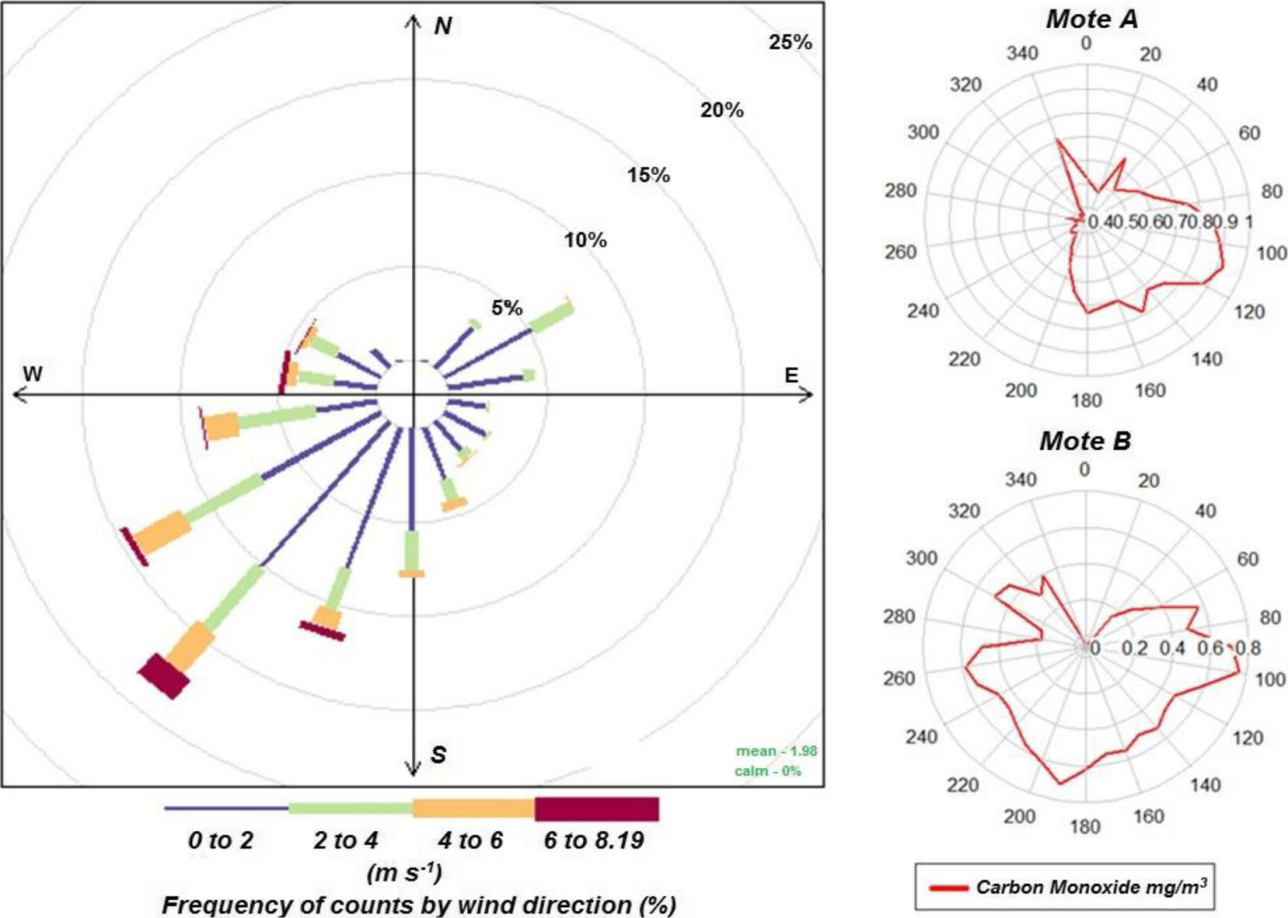
Wind rose (*left*) for the Medway meteorological sensors; CO levels of mote A in urban canyon and mote B in open space (*right*)

For this research, the wealth of traffic data available as a by-product of ITS legacy systems has been used. With reference to Fig. 2, the real-time traffic data and network information from the Split Cycle Offset Optimisation Technique, SCOOT (Hunt et al. 1981) also is captured, cleaned and processed to identify, on a cycle-by-cycle resolution, the prevailing traffic flow regime whether quiet, smooth flow, unstable or congested (Hodges et al. 2009). This congestion status is passed through to the data warehouse to build up a historic picture of traffic conditions over time and onto the common database for immediate display. A previous research has demonstrated the use of SCOOT data in estimating pollution emissions (Bell et al. 2011a) and its value in developing insights in the sources of the levels of pollution concentrations in microenvironments within street canyons, in particular relationships between traffic flow regimes (quiet, smooth flow, unstable and congested). This paper adopts the flow regimes to quantify the extent to which specific traffic states prevail in urban areas.

### Meteorological conditions and other datasets

Continuous monitoring of meteorological conditions, namely wind speed, wind direction, temperature and humidity, has been collected using three meteorological stations deployed in Medway and one station in Newcastle, in close proximity to the pervasive sensor deployment. These data, available at 10-min resolution and synchronised with the mote and traffic measurement, are required to investigate the influence of the meteorological conditions on pollutant levels. Other, static and less dynamic data was collated into the database on the understanding that they influence in some way the pollutant concentrations in urban microenvironments. These include topographical (gradients, building heights, vegetation) and road geometry (road width, orientation). The data has been obtained from the city councils.

In previous projects, this information was used, along with a version of the Operational Street Parameter Model (OSPM) to calculate the dispersion of the emissions from the SCOOT data which has been used to estimate pollution emissions using a congestion-sensitive algorithm (Bell et al 2011b).

It is known that meteorological conditions can have a marked effect on the pollution measured at any given environmental sensor. For example, increases in sunlight can alter the chemical breakdown of certain pollutants and temperature or humidity will influence the chemical reactions a pollutant will undergo after leaving a car exhaust. However, one of the most common meteorological effects is that of wind on the dispersion of the pollutant. Higher wind speeds can not only increase the dispersion of the pollutant but it can also concentrate the pollutant through the formation of vortices within an urban canyon, which can limit dispersion.

The wind will be typically heterogeneous with respect to both speed and direction and hence will have an effect on urban canyon dispersion which will systematically vary depending on the orientation of the canyon. In order to fully investigate this, it is necessary to construct a full model for urban canyon dispersion (Bright et al. 2013); however, the effect of wind direction, compared to the angle of the urban canyon, can still be observed on a more quantitative basis. This is demonstrated in Fig. 2 by plotting the average of the more chemically stable (than either NO or NO_2_) CO pollution level for measured wind speeds and direction. The urban canyon for mote A is aligned along the 170° direction with the 80°–100° direction, representing the point at which the mote would be caught in a wind-induced canyon vortex. By contrast, mote B, although is close to a road with similar alignment, is in a more open environment and exhibits a much reduced level of directionality for the pollution average by wind direction. Taken together, these figures offer tentative evidence for the systematic influence of wind direction on pollutant dispersion in the built environment over sustained periods of time (months, years) and highlight the importance of meteorological variables on the magnitude of measured pollution and prediction. This is explored more fully in Bell et al. (2011b).

In Medway, the wind variables showed no systematic variation with time of day; therefore, it is assumed that the wind variables will not have a daily systematic effect on the measured pollutant levels. This assumption will not always be justified, especially in coastal cities where the wind will have a strong, systematic variation with time of day due to the coastal winds, as was the case in earlier mote deployment in Palermo, Sicily (Galatioto and Bell 2013). Although varying wind speed and wind direction will affect the absolute levels of air pollution, it is not necessary to use the wind variables as a factor in the context of the cluster analysis performed here. This is because, it is assumed that the underlying driver for the daily profile of pollutants measured at the motes is traffic flow regimes of the traffic that govern the emissions. Therefore, the cluster analysis explores the relationships between the shape of the daily averaged profiles of traffic flow and the shape of the daily averaged pollutant concentrations measured at the motes. The static parameters are used to identify possible reasons for systematic variations between SCOOT and mote data sets. Future work will use the full range of meteorological data to more accurately determine the pollution level at each mote as derived from the SCOOT data-dependent emissions.

### Demonstrator site

**Fig. 3:**
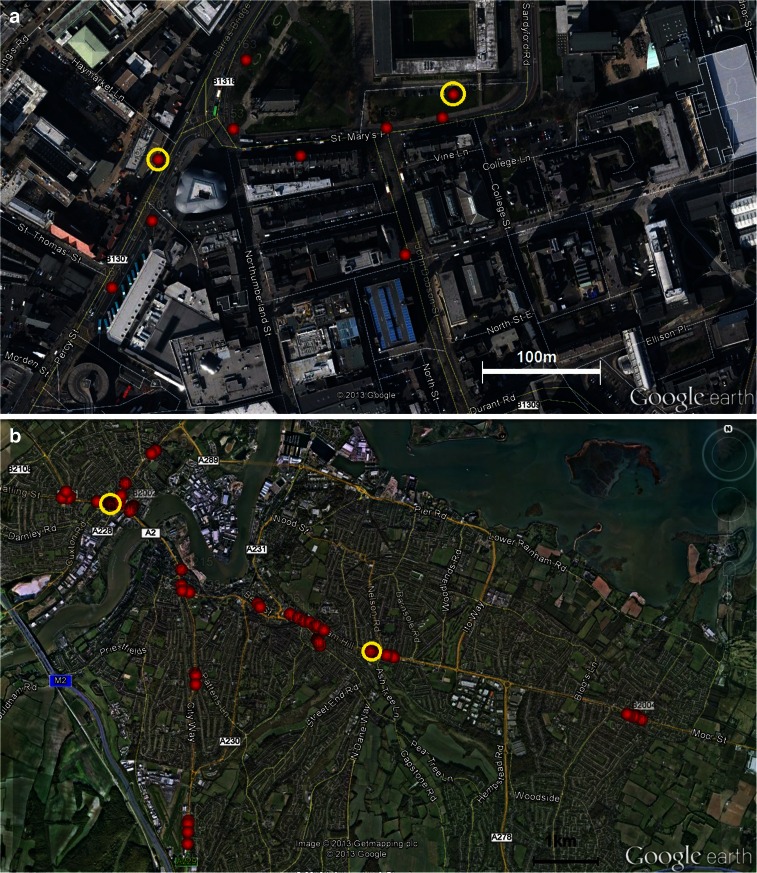
Demonstration areas in **a** Newcastle upon Tyne and **b** Medway from where data was made available for this study (*red dots* indicate mote locations, those circled in yellow are co-located with precision monitoring)

After the successful trial of 50 prototype motes deployed in Leicester (UK) (Hodges et al. 2009; North et al. 2009), the capability of the motes to monitor and characterise noise levels in urban areas and street canyons was demonstrated (Bell et al. 2009; Bell and Galatioto 2013), and preliminary work on the validation of mote data and time series of pollution concentrations in urban areas can be found in Bell et al. (2011a). Subsequently, following commercialisation of the technology by the UK company Envirowatch, over 200 commercial motes have been deployed across several UK cities, Newcastle upon Tyne (Fig. 3a), Medway (Fig. 3b) and more recently, in Perth, Liverpool and in Stockport.

Most of the mote arrays have been deployed in AQMAs, where existing background and roadside precision monitoring have continued to be used for validation of pollutant concentrations measured by co-locating one or two pervasive sensors.

### Steps in data preparation and statistical analysis

Air and noise pollution data from the motes located in Newcastle (14 motes) and Medway (108 motes) have been continuously collected during the period October–November 2011. In Medway, simultaneous measurements of traffic flow from the demand-responsive control system SCOOT were collected along with meteorological conditions. Topography (gradient) and built environment characteristics (road widths, height of buildings) were assigned to each mote which in turn was associated with a particular SCOOT link as appropriate. The initial analysis was to process the raw data, filtering for faulty records, managing missing data (using interpolation for fewer than three consecutive data records missing; otherwise, *no data dummy* assigned and ignored in the analysis), synchronising data to a master clock (in this case, the UTMC Greenwich Mean Time) and finally, averaging to 5/15/60 min to facilitate different analysis. This processing was achieved using the NUIDAP. The next step was to carry out a validation exercise to establish the level of statistical confidence in the measurements made by the motes compared with the precision monitoring system. For this validation purpose, precision measurements from an air quality monitoring site in Newcastle have been collected (https://www.dataview247.com/Default.ltr.aspx) during the same period.

Following the validation step, the *goodness* of the motes in measuring pollutants such as NO and NO_2_ was established. The patterns in the distribution of 1-min pollutant averages and characteristics of the variation in pollution concentration levels over the day depending on the mote location was investigated; the positions within canyons, with different height-to-width ratios, on different sides of the road, at various distances away from the stop-line and dynamic conditions (e.g. meteorological and traffic conditions), were explored using clustering techniques. For this particular analysis, the clustering technique used was similar to that suggested by Liao (2005). For each location and for each variable to be measured, a single normalised daily profile representing an average day was created. This gives the relative level of each variable at a given hour compared to the maximum level for an average day. Normalising the profile in this way allows comparison of daily profiles between locations which in reality will experience different absolute levels. In essence, the comparison is only concerned with the shapes of the profiles, not their absolute values. If the levels were not normalised, then the differences between profiles would be dominated by variations in the absolute level of pollution which were not only solely being driven by traffic emissions but also by the mote position, prevailing wind dispersion, etc. To form the clusters, an agglomerative hierarchical technique was used with the comparative distance between profiles being determined by the Euclidean distance between each profile. Specifically, for this work, Ward’s minimum variance algorithm (Ward 1963) was used to perform the clustering. Further analysis considered the meteorological conditions and static data as parameters to provide a richer understanding of the complexities of the processes governing concentrations and dynamic emission sources. This knowledge is vital to inform traffic managers of ways to better control traffic in urban areas to mitigate their impact on health. The next section presents the “Results and discussion”.

## Results and discussion

### Validation of sensors

**Fig. 4:**
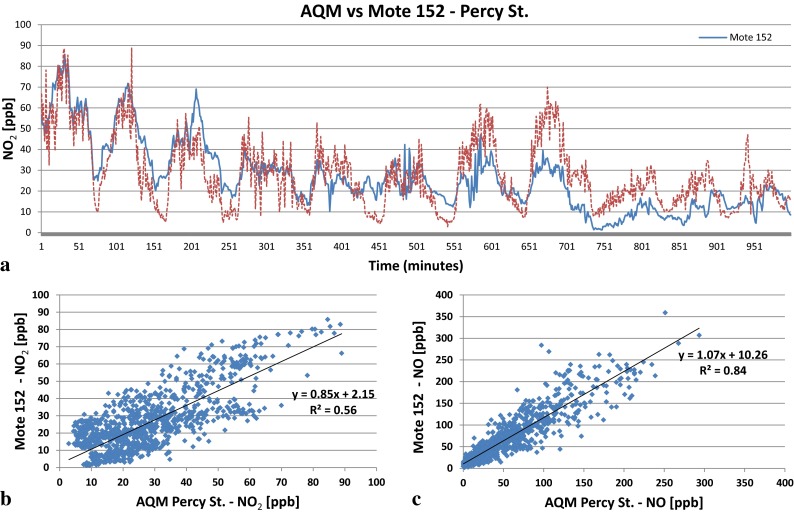
Comparisons of 15-min data monitored using a precision measurement system (AQM, Casella) plotted against mote 152 for **a** time series for NO_2_, **b** regression for NO_2_ and **c** regression for NO

For the validation exercise, 2 weeks in October 2011 of simultaneous data of mote 152 located in Percy Street in Newcastle and AQM Station has been used. As the data from the AQM Station is available only at 15-min resolution, the time series and correlation of precision against mote data has been based on 15-min averages and presented in Fig. 4 for the two common pollutants measured, NO_2_ and NO. Given that the two sensor systems are some 1.5-m apart and as such are not “breathing” the same air, variation in the data is due also to real differences in the level of pollution concentrations. Early research with the prototype motes (Bell et al. 2009, 2011a) has shown temperature and humidity effects on the chemical sensors. It is for this reason that these variables are measured and self-correction algorithms have been developed to dynamically (at 1-min resolution) correct the raw sensor measurement based on real-time measurement of temperature and humidity. Figure 4a shows that the mote 152 tracks the 15-min variation of NO_2_ concentrations which is an indication of the capability of the low-cost sensor to track changes in ambient pollution. However, as shown in Fig. 4b, the NO_2_ measurement (corrected for temperature and humidity) is more widely spread and suggests clusters in the data. There are several potential reasons for this, which illustrate the challenges faced in the particular research presented in this paper. First of all, it could be due to cross sensitivity of the NO_2_ sensor deployed in the mote to ozone. The supplier warns that the NO_2_ sensor is cross sensitive to ozone; however, it is not certain by how much (i.e. 10, 20 %, etc.) nor is there any indication whether or not the degree of cross sensitivity is linear with ozone level. This renders the need for caution in the interpretation of the NO_2_ data. The regression plot clearly shows “families” within the data. This data is not corrected for ozone sensitivity. The *upper* group with a slope that is not statistically significantly different from unity has statistically significant offset of +10 μg m^−3^ represents periods during the day when ozone levels in the atmosphere are low and therefore do not affect substantially the NO_2_ levels. The *lower* group illustrates periods of the day, usually overnight, when ozone is high and therefore the motes measure some fraction of ozone as NO_2_, thus overestimating NO_2_ levels. Underpinning this data is the classic ozone–NO_2_ relationship (Jenkin 2005) which suggests cross sensitivity as a function of ozone level. This reasoning makes the assumption that the cross sensitivity to NO_2_ of the sensor is independent of whether NO_2_ results from secondary formation or primary NO_2_, the relative amounts of which are governed by the relative proportion of petrol to diesel vehicles. Another potential reason for this scatter about the regression line could be the close proximity to temporary scaffolding during the survey period, creating local eddies which influence cooling and heating effects of shading and the dispersion in the artificial microenvironment.

Turning now to Fig. 4c, the NO from the mote correlates well with the precision measurement with an *R* ^2^ = 0.84 with slope and intercept not statistically significantly different from 1 and 0, respectively. The mote NO value is self-corrected for temperature and changes in humidity in the similar way to the NO_2_ and experiencing any localised dispersion caused by the scaffolding without displaying any data grouping seen in the NO_2_ plot. However, unlike the NO_2_ sensor, the NO sensor is not cross sensitive to ozone; therefore, it is suggested that the grouping of data seen in Fig. 4b is probably reflecting, also, the ozone cross sensitivity.

Given the cross sensitivity issues highlighted in this validation exercise, this research focussed on the analysis of NO. Based on the validation exercise presented above and given that this particular co-location is some 1.5 m distant and therefore the monitors are not “breathing” the same air, the error in measurement is about 7 %. The carbon monoxide, CO, sensors have in previous research (Bell et al. 2009) shown to give reliable data with a measurement error of about 1 % and whilst not a pollutant of concern in the UK, is a fairly stable gas and tracks traffic flow regimes (of petrol engine vehicles) in urban areas. Therefore, CO and NO levels have been analysed to provide an independent analysis to support the results of the NO_2_.

### Clustering of sensors

The first step in the analysis makes the basic assumption that the concentrations measured by the mote*s* will exhibit profiles changing from day to day consistent with its location from and the dynamics of the sources of emissions. For example, motes which are on a major urban route into a city centre may be expected to show two pollution spikes resulting from typical morning and evening peaks in traffic flow and congestion. However, not all motes are sited on two-way roads, in which case the pollution profile will depend on when in the day and for what proportion of the time in the day particular traffic flow regimes prevail. Some roads have elevated pollutant levels during the morning to coincide with the morning rush hour but are without an evening peak and therefore pollutant levels are reduced in the late afternoon. On other roads, the reverse may be true. The main objective of this analysis is to establish statistical evidence that the traffic flow regimes are the major contributors to the diurnal profiles of pollution concentrations measured at the roadside. As such, in Medway and Newcastle, from where data was collected for this current study, daily variations in meteorological conditions and topography and urban microenvironments are considered to be less dynamic and therefore second-order effects.

Adopting cluster analysis techniques, the shape of the daily pollutant concentration profile for NO and daily traffic flow profiles were analysed independently and the mote clusters compared to establish any statistically significant consistency in the two sets. The clustering of each mote was achieved by using a form of comparative metric (Liao 2005) which resulted in statistically generated clusters of motes displaying similar shapes. In this instance, the optimisation metric used was the distance between each daily profile, calculated using a simple Euclidean distance function. The matrix of Euclidean distances was then used as the input for a hierarchical agglomerative cluster using Ward’s criterion to determine the clustering.

**Fig. 5:**
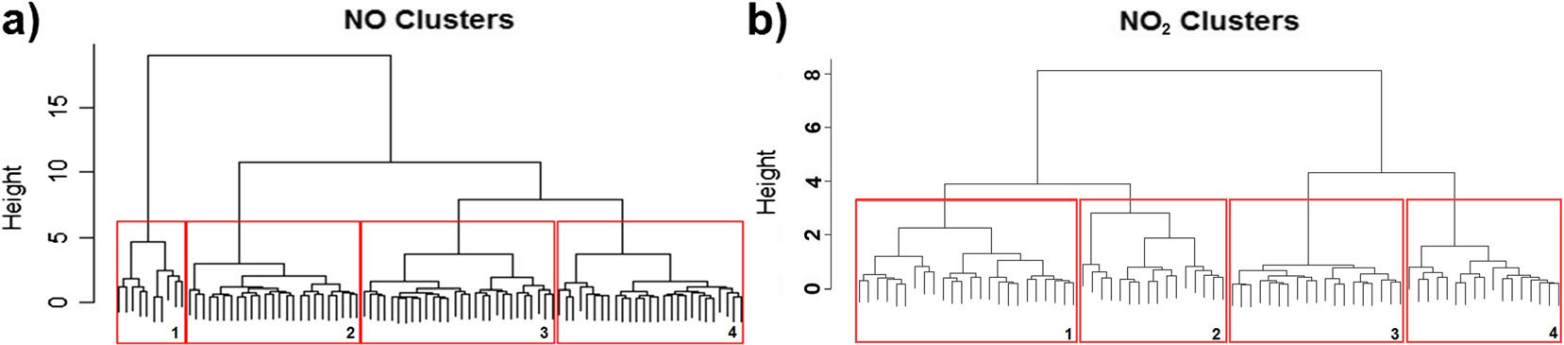
A plot of nitric oxide (a) and nitrogen dioxide (b) clusters

**Fig. 6:**
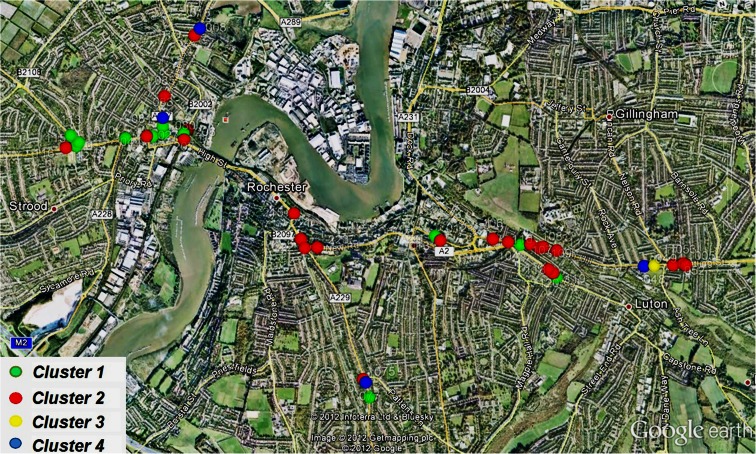
Mote sensor map representing the nitrogen dioxide clusters results in Medway

Because the absolute level of pollutant detected by the mote is heavily influenced by the position of the mote with respect to the pollutant source as well as the traffic flow regimes, it was decided to normalise each daily profile before performing the cluster analysis. Normalising the profile removes the dependence on relative distance from the pollutant source and creates a daily profile which is only dependent on the relative sizes of the pollutant values for different times of the day. After the normalisation and clustering has been performed, it can be seen that there are four natural groupings within the cluster for NO (Fig. 5a) and NO_2_ (Fig. 5b). The spatial distribution of motes based on the NO_2_ clustering is illustrated in Fig. 6 for the Medway towns deployment.

**Fig. 7:**
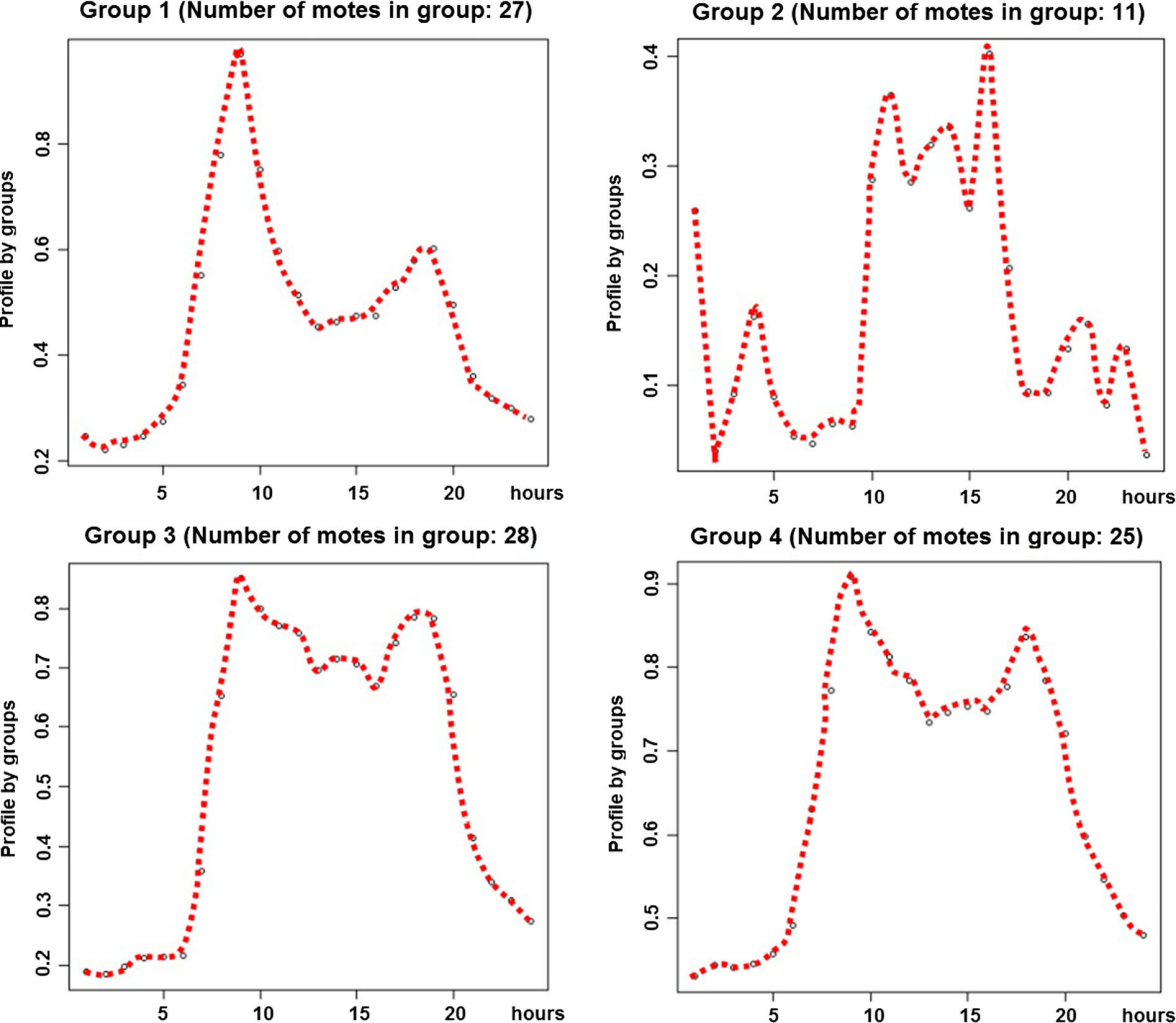
Group of daily profiles of normalised NO concentration in Medway

By averaging the daily profile within each group, the four different daily profiles around which the motes naturally cluster can be observed (Fig. 7). It can be seen that all four clusters share one feature, an increased level of pollution during the day time. However, the fine-grained structure of the daily profile is more complex with one profile exhibiting pronounced morning and evening peaks, one profile exhibiting morning and evening peaks but with an increased inter-peak level, one profile showing a comparatively reduced peak level and the final profile showing a much “noisier” daily profile.

### Discussion

#### Sensor sites characterisation

In Newcastle, only three clusters of the four identified have been associated with motes according to the cluster analysis of “Clustering of sensors” for NO levels. From looking in detail at each mote location within each cluster, it has been possible to identify specific combination of sites and characteristics associated with each cluster (Table 1).

**Table 1:**
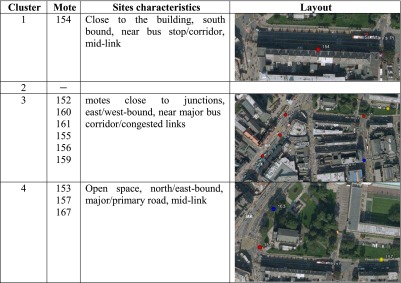
Sites characteristics in Newcastle associated with each NO cluster

Similarly in Medway, for NO, each mote location has been scrutinised within each cluster, and specific combination of site characteristics has been associated with each cluster (Table 2).

**Table 2:**
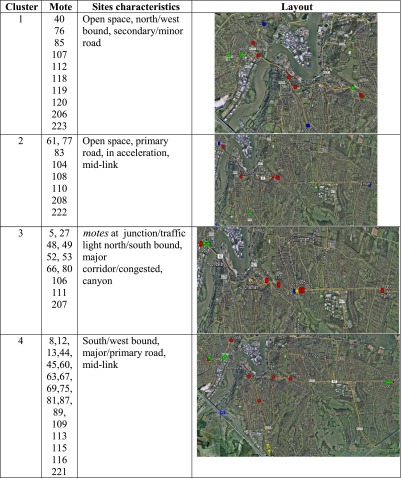
Sites characteristics in Medway associated with each NO cluster

By identifying specific combination of sites with generic features associated with each cluster, it becomes clear that clusters 3 and 4 have similar characteristics between the two demonstration sites in Newcastle and Medway, respectively. This is particularly relevant when considering that the two clusters 3 and 4 are either in close proximity to a junction and major/primary road (cluster 3) and major/primary road, mid-link (cluster 4).

In order to confirm the association of the two clusters with unstable and congested link, a preliminary analysis of the traffic status using the available traffic data from the link on which the motes are located has been carried out in the next section.

#### Influence of congestion on mote clusters

**Fig. 8:**
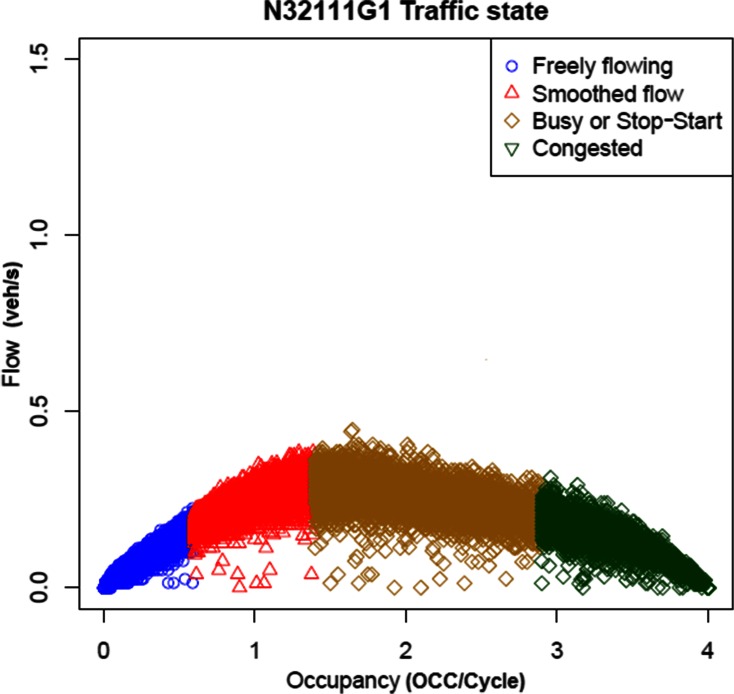
Normalised flow–occupancy graph with associated traffic status

Access to the SCOOT system in Medway enabled the collection of traffic information from the Medway network. In particular, traffic signal cycle, flow and occupancy information from each SCOOT loop in close proximity of a mote sensor has been collected during the survey period in order to characterise the link in terms of traffic status. An example of normalised flow–occupancy graph with associated traffic status is presented in Fig. 8. Where “flow” is expressed in veh/s and “occupancy” is the ratio of the number of ¼ s the SCOOT detector loop (N32111G1) is occupied during each traffic signal cycle, from OCC message from SCOOT, and the cycle time in seconds.

Adopting the same cluster analysis method used for the motes’ concentrations, more than 200 SCOOT links have been clustered into five groups, depending on the percentage and ratio between the four traffic states identified in earlier research (Bell et al. 2011b). These four states that govern the emissions, and therefore, roadside concentrations are namely free flow (state 1), smooth flow (2), unstable (3) and congested (4). These four states, respectively, were associated with “occupancy” ranges of 0 to 0.6, >0.6 to 1.3, >1.3 to 2.9 and >2.9, respectively. In Table 3, the five clusters are presented, indicating the average proportion of the time over the day (expressed as a percentage) for which each traffic state prevailed along with the number of links falling into that group.

**Table 3:** Number of links and the traffic status distribution associated to each of the five clusters

	1	2	3	4	5
State_1	43.42	98.61	90.78	75.48	2.70
State_2	33.68	1.01	7.27	17.13	0.07
State_3	15.81	0.18	1.36	3.86	0.50
State_4	7.09	0.20	0.59	3.53	96.73
num_links	10	78	67	53	3

After removing cluster 5, which is classified as an outlier due to the data being identified as likely faults in the detectors, the other four clusters can be split into two clearly identified groups. In clusters 1 and 4, where there is a medium-high presence of congestion, but at the same time characterised by a high percentage of traffic moving in state 2 (red triangles in Fig. 8), these correspond to a high volume of traffic moving at a relatively high speed. The latter is particularly representative of a traffic state (or flow regime) associated with higher NOx emissions, and for that, we are expecting to identify a correspondence between mote clusters and these two SCOOT groups.

After identification of all the motes in the vicinity of one or more SCOOT loops, mote clusters 3 and 4 have been identified as the two clusters related to SCOOT clusters 1 and 4 in more than 75 % of the cases. Below are listed the specific motes belonging to clusters 3 and 4 and identified in the vicinity of a SCOOT loop for which there is a given proportion (%) of time over the period that the link is in the state of congestion, unstable and/or smooth flow.

- Mote cluster 4
  - Motes 12 and 13 are associated with SCOOT link N34111A1 (smooth 12 %, congested 31 %).
  - Motes 60 and 69 are associated with SCOOT links N40231D1 (smooth 12 %, unstable 14 %, congested 3 %).
  - Mote 221 is associated with SCOOT links N31111E1 (smooth 27 %, unstable 25 %, congested 16 %).

- Mote cluster 3
  - Motes 40, 27 and 80 are associated with two SCOOT links N32111E1 (smooth 9 %, unstable 26 %, congested 30 %) and N32111G1 (smooth 30 %, unstable 18 %, congested 9 %, respectively).
  - Mote 66 is associated with SCOOT link N40231D1 (smooth 12 %, unstable 14 %, congested 3 %).
  - Motes 5, 48, 49, 52 and 53 are associated with two SCOOT links N37181D1 (smooth 6 %, congested 7 %) and N37121B1 (smooth 11 %, unstable 4 %, congested 3 %, respectively).

For both mote clusters, 50 % or more motes have been associated with SCOOT links, and in all the cases, the corresponding SCOOT loops were characterised by “non-free flow state” links with levels of congestion prevailing for between 3 and 30 % of the period studied.

The analysis presented so far has considered clusters firstly identified by the concentrations and secondly by the traffic flow regime with clear indication of causal links between the two. Therefore, instead of performing the clustering using two different distance matrices, the relationship between the data was more fully examined by constructing a single conceptual object using data from the collocated mote and SCOOT sensors. The object comprised only those motes and SCOOT links which were on the same position over the road link and also on the same road side. If the clustering of the motes *was* being strongly driven purely by the nearest traffic pollution source, namely the adjacent road link, then any clustering algorithm should cluster the SCOOT links and mote sensors in a statistically similar fashion. Due to the necessity for co-location, the total number of SCOOT links and motes was reduced down to 22 unique pairings.

Also, in contrast to the previous SCOOT clustering algorithm, the SCOOT data was clustered on the Euclidean distance between the normalised daily flow profiles as this is the data which is most similar conceptually to the daily pollution profiles recorded by the motes. In this case, it is expected that if the mote pollution profile was directly related to the flow/occupancy profile of the SCOOT system, then the clusters formed would be identical. NO_2_ concentrations were considered as well as CO and NO in this exercise because ozone is a global greenhouse gas which prevails across the city and NO_2_ levels are directly a function of localised emissions levels of NO which are predominantly due to traffic. This means that the cross sensitivity effect of the NO_2_ sensor to ozone is consistent for all motes in a region and therefore reflects traffic levels.

In order to compare the two populations, the same clustering technique (Euclidean distance and Ward hierarchical clustering) was used on both sets of daily profiles, the subsequent groupings were then compared using the Rand index (Rand 1971), both adjusted and normal, to assess the clusters for similarity. If the two populations of daily profiles, when clustered using the same techniques, produce identical or at least similar clustering, then this is evidence that there is an underlying relationship between the two variables.

Table 4 shows the similarity to each other of each clustering on the three main mote air pollution variables and to the SCOOT daily profile. It was found that the Rand index for the clustering between the SCOOT daily profiles and the environmental variables varied between 0.58 and 0.64, indicating that whilst the clusters were not completely independent of each other, there was no statistical justification to suggest that the classification of a mote location as belonging to a particular cluster was identical based on the use of both sets of data. In essence, the mote and SCOOT data were related but neither could be used to accurately predict the form for the daily profile of the other.

**Table 4:** The Rand index for the three pollutants and the SCOOT daily profile is shown here

	SCOOT	CO	NO	NO_2_
SCOOT	–	0.632	0.584	0.628
CO	0.632	–	0.502	0.554
NO	0.584	0.502	–	0.636
NO_2_	0.628	0.554	0.636	–

In addition, the clustering of the emitted pollutants measured at the motes showed a relatively weak dependence on each other, indicating that despite each pollutant having the same underlying sources, namely vehicle emissions, there are underlying processes, whether physical or chemical, which have first-order effect to interfere with the direct relationship between emitted pollutants and the concentrations.

Possible explanations for this compared to CO is the presence of ozone in the atmosphere affecting the ratio of NO-to-NO_2_ and consequently the ratio of both pollutants. In addition, the factors which also govern the levels of pollution at the locations of the 122 sites used in this analysis include the orientation of the road with respect to the prevailing wind, whether or not the road has a gradient, is in a canyon or affected by local microenvironments such as car parks which create opportunity for natural ventilation or bus and speed humps elevating localised emissions due to imposed interruption to traffic flows.

To more systematically investigate this, the effect of road orientation, mote position and street type for the clusters was investigated. If a systematic influence exists for each of these factors, then there will be a systematic difference depending on road orientation, mote position and street type in the daily mote and/or SCOOT profiles which may influence the allocation to a cluster.

However, the results showed that there was no statistically significant difference in any of the possible factors between paired SCOOT and mote elements when differentiated by shared membership of identical clusters. From this, we can conclude that the difference in cluster membership for SCOOT and mote profiles is being driven by something else other than the static parameters which partially govern the dispersion from the emitted pollutants. In light of this, further work is needed on the effect of meteorology and other parameters on the clustering and classification of daily profiles.

## Conclusions and future directions

In terms of estimating pollution levels, this research leads to the conclusion that it is impossible to assign to a location a pollution profile “type” based purely on the traffic flow profiles measured at that location. Although pollution levels are governed by the traffic emission and therefore the two data sets are connected, they are not identical in terms of cluster construction. It is likely that this is largely due to other environmental factors which are affecting the dispersion of the pollutant from the vehicle’s exhaust. For example, as well as the road gradient and presence of a bus stop or traffic calming hump affecting the emissions, the orientation of the road with respect to the wind direction will strongly affect the pollutant level as measured at the mote’s position.

Although the daily traffic flow profile is not sufficient, it may be possible to use other variables to successfully create identical clustering data sets which will improve the characterisation of the daily traffic profiles to such an extent that they can be successfully mapped onto the clusters derived from the daily profiles of the pollution as measured by the motes. Such variables may include street orientation, street type (canyon or open), prevailing weather or the presence of trees within the road network. By combining each of these variables with a dispersion model (OSPM or street for example), it would be possible to more completely model the dispersion and hence achieve a more accurate correlation in the groupings between SCOOT and the motes. However, this research clearly illustrates the benefits of pervasive measurement of concentrations in urban microenvironments to effectively evaluate human exposure to transport-related emissions.

## Footnotes

1 AQMAs are identified with any locations within local authority boundaries where the Air Quality Objectives (threshold limits set by the government to minimise health risk) are not likely to be achieved. Each area may encompass just one or two streets or it could be much bigger. The local authority is subsequently required to put together a plan (Local Air Quality Action Plan) to address excessive source emission in order to improve air quality in that area.
